# Transcriptome Analysis Provides a Preliminary Regulation Route of the Ethylene Signal Transduction Component, *SlEIN2*, during Tomato Ripening

**DOI:** 10.1371/journal.pone.0168287

**Published:** 2016-12-14

**Authors:** Rui-Heng Wang, Xin-Yu Yuan, Lan-Huan Meng, Ben-Zhong Zhu, Hong-liang Zhu, Yun-Bo Luo, Da-Qi Fu

**Affiliations:** Laboratory of Food Biotechnology, College of Food Science and Nutritional Engineering, China Agricultural University, Haidian District, Beijing, China; Institute of Genetics and Developmental Biology Chinese Academy of Sciences, CHINA

## Abstract

Ethylene is crucial in climacteric fruit ripening. The ethylene signal pathway regulates several physiological alterations such as softening, carotenoid accumulation and sugar level reduction, and production of volatile compounds. All these physiological processes are controlled by numerous genes and their expression simultaneously changes at the onset of ripening. *Ethylene insensitive 2* (*EIN2*) is a key component for ethylene signal transduction, and its mutation causes ethylene insensitivity. In tomato, silencing *SlEIN2* resulted in a non-ripening phenotype and low ethylene production. RNA sequencing of *SlEIN2*-silenced and wild type tomato, and differential gene expression analyses, indicated that silencing *SlEIN2* caused changes in more than 4,000 genes, including those related to photosynthesis, defense, and secondary metabolism. The relative expression level of 28 genes covering ripening-associated transcription factors, ethylene biosynthesis, ethylene signal pathway, chlorophyll binding proteins, lycopene and aroma biosynthesis, and defense pathway, showed that *SlEIN2* influences *ripening inhibitor* (*RIN*) in a feedback loop, thus controlling the expression of several other genes. *SlEIN2* regulates many aspects of fruit ripening, and is a key factor in the ethylene signal transduction pathway. Silencing *SlEIN2* ultimately results in lycopene biosynthesis inhibition, which is the reason why tomato does not turn red, and this gene also affects the expression of several defense-associated genes. Although *SlEIN2*-silenced and green wild type fruits are similar in appearance, their metabolism is significantly different at the molecular level.

## Introduction

The gaseous phytohormone ethylene (C_2_H_4_) is essential for developmental and physiological regulation in a variety of higher plants [1] and its effect is more obvious during the ripening process of climacteric fruits [2]. Physiological and molecular genetic analysis have uncovered a pathway for ethylene signal transduction, which is evolutionarily conserved from the receptors of the endoplasmic reticulum membrane to transcription factors in the nucleus [3,4,5,6]. The ethylene receptor (ETR), localized on the endoplasmic reticulum, negatively regulates the ethylene signal transduction occurring upstream [4,7]. Several years later, studies in root hair cells by Dong et al (2010) indicated that receptor ETR1 also localizes at the Golgi network and only partially at the ER [8]. So far seven *ETR* genes of tomato (*Solanum lycopersicum*) have been cloned, *SlETR1*, *SlETR2*, *NR*, *SlETR4*, *SlETR5*, *SLETR6*, and *SlETR7* [9,10,11,12], present different expression patterns during tomato development [13]. *Arabidopsis thaliana* constitutive triple response 1 (*CTR1*), which is a Raf-like protein kinase [5], has multiple homologs in tomato. Some CTR1-like proteins can interact with ETR proteins and negatively regulate signal transduction [14]. In addition, *SlCTR1* is sensitive to exo-ethylene treatment and its expression increases during tomato development [15].

In *A*. *thaliana*, ethylene insensitive 2 (EIN2), also located in the ER, is a critical positive regulator in the ethylene signal pathway and its C-terminal domain (CEND) can be cleaved and transferred to the nucleus, stabilizing another positive regulation protein, ethylene insensitive 3 (EIN3) [3,16]. ETR1 interacts with EIN2, in which Ser645 is phosphorylated by CTR1 to block the cleavage of CEND and its transfer to the nucleus [6,17,18]. EIN2 is the only protein whose loss-of-function mutation results in complete ethylene insensitivity in the ethylene signal transduction pathway between the nucleus and cytoplasm [16]. Two F-box proteins, ETP1 and ETP2, destroy EIN2 through the ubiquitin pathway [19]. In turn, EIN2’s cleaved CEND can inhibit the expression of the F-box genes *EB1* and *EBF2*, disrupting the accumulation of EIN3 by recognizing their 3′-untranslated regions and transferring them to the P-body. This is accompanied by an exoribonuclease, EIN5, acting in the cytoplasm at translation level [20,21]. Using virus induced gene silence (VIGS), *SlEIN2* can be silenced in tomato plants, significantly suppressing fruit ripening [22]. Silencing only one of the functionally redundant *SlEILs* (coding for EIN3-like proteins) did not produce significantly non-ripening phenotypes [23]. After ethylene insensitive-like (EIL) proteins bind to the promoter regions of ethylene response elements (EREs), the ethylene response factors (ERFs) are able to bind the GCC-box, a conserved sequence of ethylene response genes, and activate the ethylene-induced pathogenesis-related genes [24,25,26]. In banana, which is also a climacteric fruit, the GCC-box motif is homologous to the cis-acting elements of *MaEXP1*, suggesting that some *ERFs* might have a role in fruit softening [27].

Fruit ripening is a complicated process, including the accumulation of volatile components, flavonoids formation, pectin degradation, and carotenoid biosynthesis [24,28,29,30]. These diverse processes are regulated by numerous transcription factors and signal transduction pathways [24], among which the ethylene signal pathway is typically found. Unlike the functionally redundant ETRs, CTRs, EILs, and ERFs, the uniqueness of EIN2 might enable it to participate in many ethylene-related metabolic pathways, which probably contributes to the complete ethylene insensitivity resulting from a mutation in the functional domain of *SlEIN2*. Previous studies have shown that non-ripening tomatoes were obtained by silencing *SlEIN2* through VIGS, and recently a new paper from Gao et al (2016) showed that *in yellow-fruited tomato 1*(*yft1*) mutant, lower expressed of *EIN2* would lead to impaired ethylene biosynthesis and perception, as well as abnormal carotenoid production [22,31]. However, no study has assessed how *SlEIN2* regulates fruit ripening at the gene level. Therefore, the present study aims to provide a preliminary analysis of *SlEIN2* regulation during fruit ripening using RNA sequencing (RNA-seq). The results showed that silencing *SlEIN2* leads to significant changes in the expression of a large number of genes involved in chlorophyll binding proteins, ethylene biosynthesis, lycopene production, defense, etc., as *SlEIN2* and the ethylene signal pathway critically upregulate several transcription factors in a feedback loop. The study of *SlEIN2* also increases the knowledge on the molecular mechanisms regulating fruit ripening by signal transduction pathways.

## Materials and Methods

### Plant material and growth conditions

*Ailsa Craig* tomato seeds preserved in our laboratory were sown in commercially available tomato-cultivation soil and grown in a chamber at 25 ± 2°C, with a relative humidity of 75% and under a light: darkness cycle of 16:8 h, regulated by fluorescent lamps. Tomato plants were watered with a nutrient solution once a week.

### Tobacco rattle virus (TRV)-*SlEIN2* vector construction

Vectors used VIGS are based on the TRV pTRV1 and pTRV2 (Liu Y et al., 2002). We adopted In-fusion^®^ (Clontech, Nanjing, China), a new cloning technique that does not require T4 DNA ligase and the insertion of a silencing fragment, and has high ligation efficiency. To generate pTRV2-*SlEIN2*, pTRV2 plasmids were first linearized through digestion with *Eco*RI and *Bam*HI. As the reverse insertion of the silencing fragment can improve silencing efficiency [22], a 348-bp *SlEIN2* fragment was amplified using the forward primer 5′-TAAGGTTACCGAATTCCCTGAATTGGAGCTGTAC-3′, which included a *Bam*HI adaptor (underlined) and the reverse primer 5′- GCTCGGTACCGGATCCTGGAAATGTCCCTGTAGG-3′, which included an *Eco*RI adaptor (underlined). The resulting product was cloned into pTRV2 using the In-fusion^®^ kit and following the manufacturer’s instructions.

### *Agrobacterium tumefaciens* infiltration

The vectors pTRV1- and pTRV2-*SlEIN2*, and the control vectors pTRV1 and pTRV2, were transformed into two sets of the *A*. *tumefaciens* strain GV3101 and cultured at 200 rpm at 28°C in the Luria—Bertani (LB) medium containing 10 mM 2-N-morpholino ethanesulfonic acid (MES) and 20 mM acetosyringone (AS), with 50 μg/mL of kanamycin, gentamycin, and rifampicin antibiotics. After 16 h, both sets of A. *tumefaciens* cells were centrifuged and resuspended in the infiltration buffer (10 mM MES, 200 μM AS, 10 mM MgCl_2_; pH 5.6), until their OD_600_ ranged between 4 and 7. Bacterial suspensions were set aside for 3–4 h and then combined at a 1:1 ratio, before their infiltration in tomato plants’ using a 1-mL syringe. Ten days after pollination, the carpopodium of tomato plants was perforated and 50–100 μL bacterial solution were infiltrated at the wound site.

### RNA isolation and real-time quantitative PCR

Five days after breaker (BK), the orange ripe (OR) pTRV and pTRV-*SlEIN2*-inoculated, only the green area sampled, fruits were collected, each type including 6 fruits. Mature green (GM) fruits from another control group were also collected to evaluate differences between gene-silenced and authentic unripe fruits. Control and gene-silenced fruits were stored at -80°C before use and their total RNA was isolated using the RNeasy^®^ Mini Kit (Qiagen, Hilden, Germany). Unwanted genomic DNA was digested using DNase I (Tiangen Biotech Co., Beijing, China). The concentration and purity of RNA were measured in a NAS-99 spectrophotometer (ATCGene Inc., New Jersey, United States). The RNA integrity estimated through gel electrophoresis showed a 28S/18S brightness ratio of approximately 2:1. Complementary DNA was then synthesized from 2 μg RNA using the *TransScript*^®^ II One-Step gDNA Removal and cDNA Synthesis SuperMix Kit (Transgen Biotech Co., LTD., Beijing, China) with oligo(dTs). Virus vectors were detected by PCR using the EasyTaq PCR SuperMix (Transgen Biotech Co., LTD), coat protein, and pTRV-RNA2 specific primers. Amplifications were performed in a Bio-Rad (Hercules, CA, United States) thermocycler under the following conditions: 94°C for 3 min, followed by 30 cycles at 94°C for 30 s, 55°C for 30 s, and 72°C for 40 s.

Quantitative PCR (qPCR) was performed in the Bio-Rad CFX96 thermocycler using the TransStart Top Green qPCR SuperMix (Transgen Biotech Co., LTD) for 5 min at 95°C, followed by 40 cycles of 15 s at 95°C and 30 s at 60°C. Changes in fluorescence intensity were monitored in each cycle. Three biological replicates, each including two mixed fruits, were included in the PCR and expression levels were determined relatively to that of *Actin* (*ACT1*), which was used as the internal control, and analyzed using the 2^- ΔCt^ method [32]. All primers used are listed in S1 Table.

### RNA sequencing and assembly of RNA transcripts

Total RNA was isolated from green pTRV-*SlEIN2* samples and two groups of pTRV fruits (biological replicates were the same as in qPCR). Total RNA concentration was measured in the NAS-99 spectrophotometer (ATCGene Inc.), and an RNA integrity number ≥ 7.0 was confirmed through gel electrophoresis. Messenger RNA was then enriched using oligo(dTs) coupled with magnetic beads, before being cut into 300-bp fragments (Novogene, Tianjing, China). Complementary DNA libraries were obtained using random hexamers to synthesize the first strand, and adopting DNA polymerase I and dNTPs to generate the second strand. Synthesized cDNA was then purified, its ends were repaired, and adaptors were ligated. After libraries’ preparation, 150-bp pair-end sequencing was performed on an Illumina^®^ Hiseq PE150 (Illumina, Inc., Beijing, China), generating 6 G raw data for each pair-end sequencing.

Raw reads were quality checked and trimmed using cutadapt (version 1.10, https://pypi.python.org/pypi/cutadapt/) and FASTX-Toolkit (version 0.0.13.2 http://hannonlab.cshl.edu/fastx_toolkit/download.html). After removing barcode and adaptor sequences, the resulting clean reads were checked for quality using the Q < 20 threshold. All clean reads were deposited in the National Center for Biotechnology Information (NCBI) Sequence Read Archive (http://www.ncbi.nlm.nih.gov/sra/) under the accession number SRP076745. Clean reads within each library were aligned with the tomato reference genome (version SL2.50, ftp://ftp.sgn.cornell.edu/tomato_genome) using TopHat (version 2.0.8, http://ccb.jhu.edu/software/tophat/index.shtml). Reads with less than two mismatches were used to construct transcripts using Cufflinks (version 2.0.2, http://cole-trapnell-lab.github.io/cufflinks/). Genes in pTRV-*SlEIN2* and pTRV-GM or pTRV-OR fruits were considered as differentially expressed genes (DEGs) if |fold-change| ≥ 2 and *Q*-value < 0.05.

### Gene Ontology (GO) enrichment analysis

GO enrichment analysis was performed using GO-TermFinder (version 0.86, http://search.cpan.org/dist/GO-TermFinder/) based on DEGs, gene identity in the Sol genomics network database to GO terms, gene association, and GO libraries (http://geneontology.org/page/downloads). The threshold of the corrected *P*-value was 0.05, and genes were classified into the following classes: cellular component, biological process, and molecular function.

### Kyoto Encyclopedia of Genes and Genomes (KEGG) pathway enrichment analysis

Fasta format files containing DEGs cDNA or protein sequences were obtained using Perl scripts and KEGG enrichment analysis was then performed in KOBAS (version 2.0, http://kobas.cbi.pku.edu.cn/download.do), based on native blast tools and organism annotation libraries. KEGG pathways with a corrected *P*-value < 0.05 were analyzed.

## Results and Discussion

### Silencing *SlEIN2* produced non-ripening phenotype and differential expression of several thousand genes

Ethylene regulates several plant physiological activities including development, senescence, flowering, and fruit ripening through signal transduction pathways. As *SlEIN2* is an important component of signal transduction, mutations occurring in this gene will effectively block the signal transduction pathway, resulting in plant insensitivity to ethylene. To understand the role of *SlEIN2* in the development and ripening of tomato, we obtained *SlEIN2*-silenced fruits and analyzed their DEGs using RNA-seq.

**Fig 1:**
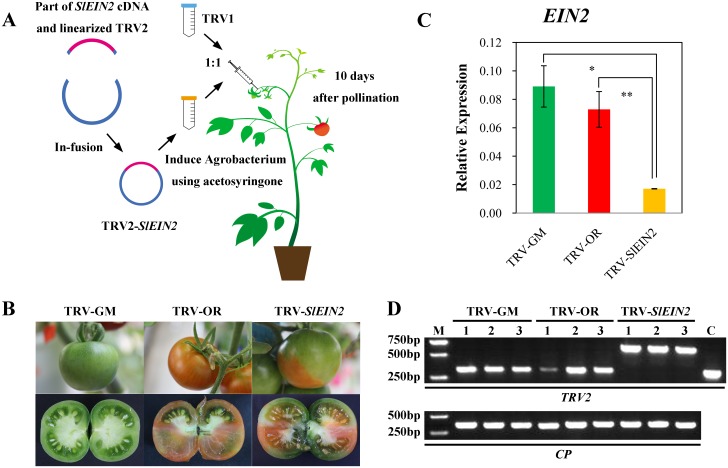
Acquisition of TRV-*SlEIN2* fruits. (A). Main phases of VIGS. (B). Non-ripening phenotype of attached and dissected TRV-*SlEIN2* tomato fruit compared with the two control groups. (C). qPCR analysis of *SlEIN2* expression in VIGS and control fruits. The error bar indicates the standard deviation, based on three biological replicates. Asterisks indicate significant differences, according to Student’s *t*-test (***, *P* < 0.001). (D). pTRV transmission in fruits. M is the 2 kb marker, and C is the control, using the pTRV2 plasmid as template. 1, 2, and 3 represent the three biological replicates.

According to the sketch presented in Fig 1A, a mixture of *A*. *tumefaciens* GV3101 cultures containing pTRV1- and pTRV2-*SlEIN2* or pTRV1 and pTRV2 constructs in a 1:1 ratio, were needle-injected into the carpopodium of wild-type *Ailsa Craig* tomato fruits 10 days after pollination. The phenotype on the fruit was observed at 5 days after breaker stage. The *SlEIN2*-silenced fruits were still green whereas the non-*SlEIN2*-silenced and control fruits were red (Fig 1B). The phenotype presented by *SlEIN2*-silenced fruits was consistent with our previous study [22]. They remained green, i.e., they were not ripe, and their appearance was very similar to that of pTRV-injected GM fruits.

To confirm *SlEIN2* gene silencing at the molecular level, primers specific to the *SlEIN2* genes outside the region targeted for silencing were designed and used in qPCR. Results evidenced a 76% reduction in *SlEIN2* transcripts in silenced fruits in relation to pTRV-injected OR fruits. The expression of *SlEIN2* in pTRV-injected GM differed from that in pTRV-*SlEIN2* fruits, the latter only accounting for 19% of the former (Fig 1C). Consideringan average of ~80% reduction of target endogenous mRNA is normally achived using TRV-VIGS, the samples were feasible for futher studies and analyses [33,34]. As the level of *ACT1* transcript was similar in tissues infected with pTRV-*SlEIN2* and control vectors, *SlEIN2* seems to play an important role in the ethylene signal transduction pathway controlling fruit ripening.

**Fig 2:**
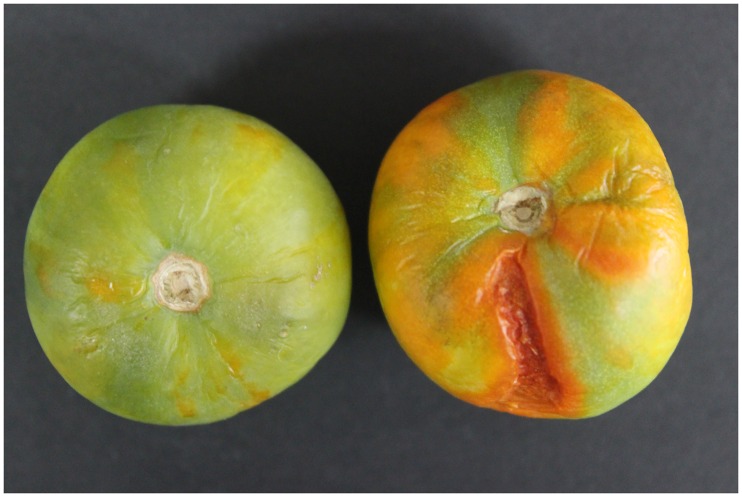
Phenotype of *SlEIN2*-silenced tomato three months after picking, BK+5 phase was initiated.

It was reported that *SlEIN2* expression reaches its peak at the GM stage, and is reduced after BK, during ripening [35]. The massive accumulation of phosphorylated *SlEIN2* in the GM stage is probably related to the upcoming respiration peak and vast changes in fruit substance and color. When fruit development reached the breaker stage, there was few EIN2 in VIGS fruits, and thus interrupted fruits ripening (Fig 2). The correct size of bands shown in Fig 1D evidenced pTRV was well transmitted to the fruits.

To understand the molecular mechanism of *SIEIN2* regulating tomato fruit ripening, *SlEIN2*-silenced and control fruit samples (pTRV-GM and pTRV-OR) were analyzed by RNA-seq. All clean reads generated in the sequencing experiment were mapped and aligned with the tomato reference genome (Table 1) Within each file, 79.56 ± 0.62% of the reads were uniquely aligned, suggesting that sequencing results were relatively stable. Discarded multiple-mapped reads (0.60 ± 0.07% of total mapped reads) and the almost uniform 42% GC-contents of sequences are not shown. Selected DEGs (SDEGs) regarding pTRV-GM/pTRV-*SlEIN2* and pTRV-OR/pTRV-*SlEIN2* fruits are listed in S2 Table.

**Table 1:** Summary of clean read counts and percentage of unique mapped reads.

Sample	Clean reads left/right	Left unique mapped	Right Unique mapped	Unique alignment
TRV-*LeEIN2* 1	20,795,992 (100%)	19,032,879 (91.52%)	17,273,527 (83.06%)	16,596,614 (79.81%)
TRV-*LeEIN2* 2	21,042,397 (100%)	19,329,124 (91.86%)	17,422,732 (82.80%)	16,772,627 (79.71%)
TRV-*LeEIN2* 3	22,409,355 (100%)	20,533,360 (91.63%)	18,780,469 (83.81%)	18075341 (80.66%)
TRV-GM 1	22,011,906 (100%)	20,122,103 (91.41%)	18301383 (83.14%)	17,594,909 (79.93%)
TRV-GM 2	22,675,897 (100%)	20,747,156 (91.49%)	18,765,171 (82.75%)	18,053,232 (79.61%)
TRV-GM 3	22,025,764 (100%)	20,100,048 (91.26%)	18,052,961 (81.96%)	17,378,831 (78.90%)
TRV-OR 1	23,938,433 (100%)	21,985,222 (91.84%)	19,547,355 (81.66%)	18,793,997 (78.51%)
TRV-OR 2	27,028,002 (100%)	24,746,795 (91.56%)	22,246,381 (82.31%)	21,423,896 (79.27%)
TRV-OR 3	21,571,779 (100%)	19,700,720 (91.33%)	17,838,181 (82.69%)	17,177,796 (79.63%)

1, 2, 3, biological replicates.

**Fig 3:**
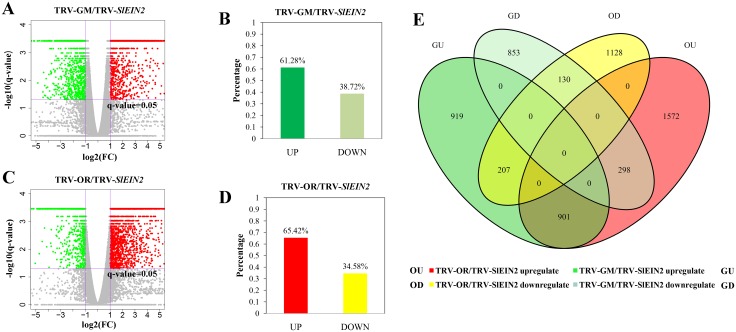
Analyses of differential expressed genes. (A, C). Volcano diagrams of DEGs. Spots above the threshold line (*Q*-value = 0.05), indicate that differences are significant. Genes whose expression was less than a half than that displayed in the control group for *Q*-value < 0.05 are displayed in the green area, while those whose expression was more than two-fold that of the control group are displayed in the red area. Genes in the grey area were neither over- or under-expressed. (B, D). Percentage of up/down regulated SDEGs. SDEGs were screened in the green and red areas. (E) Venn diagram showing the numbers of non-overlapped and overlapped SDEGs in the four conditions tested. OU and OD separately means upregulated and downregulated TRV-OR/TRV-*SlEIN2* SDEGs. GU and GD represents upregulated and downregulated TRV-GR/TRV-*SlEIN2* SDEGs respectively.

A considerable number of genes changed their expression when there was a deficiency in *SlEIN2* (Fig 3A and 3C). In the *SlEIN2*-silenced fruits, 61.28% of the SDEGs were upregulated and 38.72% were downregulated compared to the GM control group (Fig 3B). Similarly, in OR fruits, 65.42% of the SDEGs were upregulated and 34.58% were downregulated in pTRV-*SlEIN2* fruits (Fig 3D). Whereas 901 genes were upregulated in both OU and GU, only 130 genes were downregulated in OD and GD (Fig 3E). These results indicate that silencing *SlEIN2* enhances the expression of more genes.

### Classifying SDEGs through GO and Pathway enrichments

**Fig 4:**
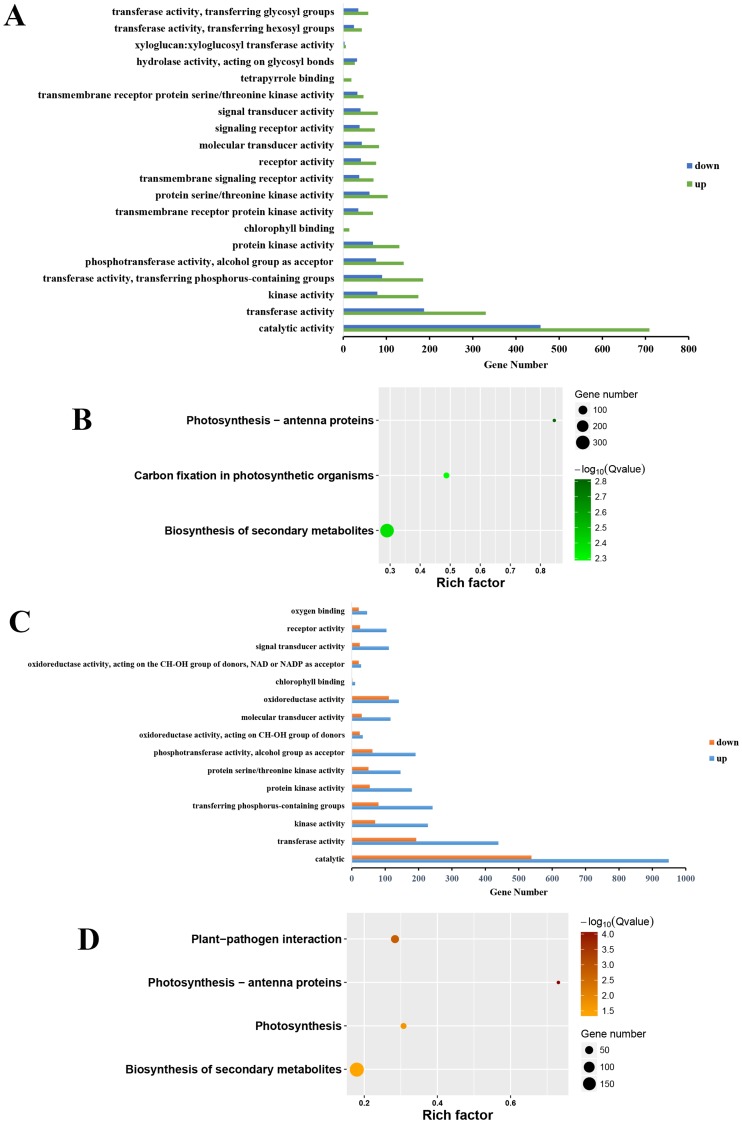
Molecular function and pathway enrichment analysis of SDEGs. (A). Molecular function of TRV-GM/TRV-*SlEIN2* SDEGs, considering a corrected *P*-value < 0.05. The X axis indicates the gene number, and Y represents classification. (B). Top 3 pathway enrichment of TRV-GM/TRV-*SlEIN2* SDEGs, with a *Q*-value < 0.05. Rich factor means the number of gene from SDEGs/all gene numbers, in a pathway. (C, D). Molecular function and top 4 pathway enrichment of TRV-OR/TRV-*SlEIN2* SDEGs. All the graphic descriptions and parameters are identical to TRV-GM/TRV-*SlEIN2*.

Gene ontology was successfully annotated in molecular function using GO-TermFinder (Fig 4A and 4C), while cell component and biological process not, since SDEGs were not significantly enriched in the two category. A diagram showing molecular functions’ connection is provided in S1 Fig. Two groups were highly enriched: catalytic and transfer activities. The concentration of catalytic proteins such as l-aminocyclopropane-l-carboxylic acid synthase (ACS), which biosynthesizes the precursor of ethylene, and the cell wall decomposition-related enzyme polygalacturonase, drastically changes during fruit ripening [30]. Transfer proteins, such as UDP-glucosyltransferases, which modify anthocyanins and flavonoids by glycosylation increasing their polarity, water solubility [36], and stability, are responsible for fruit ripening, and chlorophyll (Chl) a/b binding proteins, form Chl-protein complexes [37] that take part in photosynthesis.

In TRV-GM/TRV-*SlEIN2*, SDEGs in the class of Ser/Thr kinase were enriched while in TRV/TRV-*SlEIN2* OR fruits they were not, suggesting that most of these genes might not be regulated by *SlEIN2*. Most SDEGs of TRV-OR/TRV-*SlEIN2* exclusively related to redox activities were enriched. NAD/NADP are representative coenzymes in several metabolic activities, better known for tricarboxylic acid cycle and fatty acid oxidation. The genes enriched in NAD/NADP include several unannotated dehydrogenases and respiratory burst oxidases that are NADPH-oxidase homologs and related to plant defense [38], some acyl-CoA reductases and fatty acid oxidases that alter the composition of aroma volatiles, and the electron carrier ferredoxin (S2 Table). In addition, most genes in this class were upregulated in VIGS fruits compared to TRV-OR fruits, suggesting that they might prevent fruit ripening.

Results of the KEGG pathway enrichment analyses showed that *SlEIN2* plays a role in regulating the accumulation of chlorophyll binding proteins (Fig 4B and 4D). Light-harvest chlorophyll binding proteins (LHCPs) and chlorophyll are destabilized during fruit senescence through the regulation of staygreen (SGR) [39]. The significant enrichment of the SDEGs in the class of photosynthesis-antenna proteins indicated that LHCPs expression in *SlEIN2*-silenced fruits differed from both GM and OR fruits. Differences in carbon fixation (dark reaction) between pTRV GM and pTRV-*SlEIN2* fruits were found and pTRV OR/pTRV-*SlEIN2* SDEGs were enriched on photosynthesis (light reaction can be visualized in the link in S3 Table. Thus, although VIGS fruits were green, the genes they expressed in carbon fixation were similar to those of OR fruits, suggesting that *SlEIN2* influenced light reactions but not dark reactions in photosynthesis. In addition, silencing *SlEIN2* altered the activities of genes associated with anti-pathogen, such as the respiratory burst oxidase homolog Solyc03g117980, whose fold change was nearly 4.6 in TRV-OR/TRV-*SlEIN2* (S2 Table). Although several defense-associated genes were differentially expressed between VIGS and GM fruits, these differences were not significant according to KEGG pathways.

### Ripening-associated transcript factors are influenced by *SlEIN2* silencing

In order to verify the consistency of the RNA-seq results and the gene expression pattern in tomato fruit, 28 differentially expressed and ripening-associated genes were selected from RNA-seq results, and verified by qPCR using RNA-seq materials as template. Gene selection was based on SDEGs statistics (S2 Table) and genes identification and description are listed in Table 2.

**Table 2:** SGN ID and discription of genes detected in qPCR.

Gene	SGN ID	Discription
*EIN2*	Solyc09g007870	Ethylene insensitive 2
*RIN*	Solyc05g012020	MADS-box transcription factor MADS-RIN
*TDR4*	Solyc06g069430	FRUITFULL-like MADS-box 1
*NOR*	Solyc10g006880	NAC domain protein
*ACS2*	Solyc01g095080	1-aminocyclopropane-1-carboxylic acid synthase-2
*ACS4*	Solyc05g050010	1-aminocyclopropane-1-carboxylic acid synthase-4
*ACO1*	Solyc07g049530	1-aminocyclopropane-1-carboxylate oxidase 1
*ACO3*	Solyc09g089580	1-aminocyclopropane-1-carboxylate oxidase 3
*ETR4*	Solyc06g053710	ethylene receptor homolog
*EIL3*	Solyc01g096810	Ethylene insensitive 3 class transcription factor
*ERF2*	Solyc09g075420	Ethylene responsive transcription factor 2b
*AP2a*	Solyc03g044300	AP2-like ethylene-responsive transcription factor
*CP26*	Solyc06g063370	Chlorophyll a-b binding protein 1A
*CP29*	Solyc09g014520	Chlorophyll a-b binding protein 6A
*CAB13*	Solyc07g063600	Chlorophyll a-b binding protein 13
*GLK2*	Solyc10g008160	Transcription factor (Fragment)
*SGR1*	Solyc08g080090	Senescence-inducible chloroplast staygreen protein 2
*PSY1*	Solyc03g031860	Phytoene synthase 1
*PDS*	Solyc03g123760	Phytoene desaturase
*ZDS*	Solyc01g097810	Zeta-carotene desaturase
*PMEU1*	Solyc03g123630	Pectinesterase
*PG2A*	Solyc10g080210	Polygalacturonase A
*PL1*	Solyc03g111690	Pectate lyase
*TSRF1*	Solyc09g089930	Ethylene responsive transcription factor 1a
*PR5*	Solyc08g080670	Osmotin-like protein
*PR10*	Solyc09g090990	Major allergen Mal d 1
*LoxC*	Solyc01g006540	Lipoxygenase
*ADH2*	Solyc06g059740	Alcohol dehydrogenase 2

**Fig 5:**
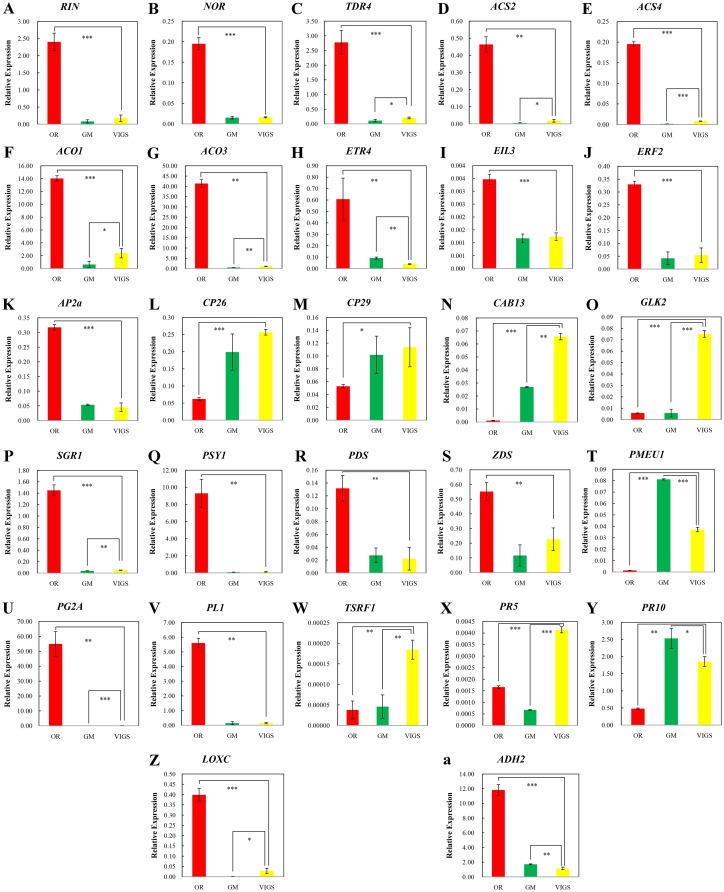
Relative expression of 28 genes covering several aspects associated with ripening. The error bar indicates the standard deviation, based on three biological replicates. Asterisks indicate significant differences, according to Student’s *t*-test (*, *P*<0.05, **, *P*<0.01, ***, *P*< 0.001).

Ripening inhibitor (RIN), a member of MADS (named by four transcription factors: MCM1, AG, DEFA and SRF) family, is an indispensible and well-known regulator of tomato fruit ripening which positively regulates gene expression by directly binding to its promoter resulting in other transcription factors expression, ethylene production, cell wall decomposition, aroma variation, and *RIN* expression [40]. The self-regulated transcription factors seems have no upstream regulator, but the ethylene signal pathway is essential for promoting the expression of *RIN* [41], suggesting that the production of ethylene is probably auto-regulated through *RIN*, and that the initial ripening is induced by a developmental factor [42]. When *SlEIN2* was repressed, the expression of *RIN* decreased drastically (Fig 5A–5C), along with its target genes, *Non ripening* (*NOR*) and *Fruitful 1* (*TDR4*) [40]. *Rin* expression was at least two-fold lower in TRV-*SlEIN2* than in OR fruits, but not significantly differs from it in GM fruits. The NOR is a ripening regulator of the NAC (named by three transcription factors: NAM, ATAF and CUC) family, whose mutation causes a green phenotype compared to the wild type at red ripe stage [43] whereas TDR4 is another transcription factor of the MADS family. It interacts with *RIN* to regulate the accumulation of lycopene and lipid metabolism during ripening. After silencing *TDR4*, ripe fruits remained orange whereas wild type were red [44]. Still, pTRV-*SlEIN2* was green for more than three months, unlike *rin-* and *nor*-silenced fruits, which are yellowish and yellowish-orange [45]. In summary, *SlEIN2* affects fruit ripening mainly by affecting RIN expression.

### *SlEIN2* silencing blocked the self-promotion of ethylene biosynthesis and gave an effect to other components in ethylene signal pathway

Several genes involved in ethylene biosynthesis are direct RIN targets, including *ACS2*, *ACS4*, and *E8* [41]. The expression of *ACS2* and *ACS4* was four times lower in pTRV-*SlEIN2* than in OR fruits (Fig 5D and 5E). In addition, *ACO1* and *ACO3* gene expressions were also significantly reduced when *SlEIN2* was silenced (Fig 5F and 5G). Studies have confirmed that *Rin* indirectly regulates the expression of *ACO1* [19,46], which is a direct target of *HB-1*; HB-1 is directly regulated by *RIN* [41,47]. The promoter of *ACO3* is the target of ERF2 [48], an ethylene responsive component at the end of the signal pathway. Thus, the abrupt drop in the expressions of these genes might have been sufficient to reduce ethylene production in gene-silenced fruits.

ETR is a negative regulation component upstream of *SlEIN2*. Tomato has seven ETRs, some of which are relatively stable while others differ from leaf to reproductive tissues [13]. The levels of *SlETR4* and *SlETR5* increase significantly as fruits mature and ripen, but only *SlETR4* changes in response to ethylene treatment [13,49]. Ethylene can enhance the mRNA level of *SlETR4*, while the protein level was on opposite. [49]. It appears that mRNA of *SlETR4* could inhibite its translation. Our study showed that silencing *SlEIN2* reduced *SlETR4* expression, as a result of inhibiting ethylene biosynthesis (Fig 5H), and this probably would promote the accumulation of ETR4 receptors When EIN2 decreased, *SlEIL3* was downregulated (Fig 5I), and the level of EIL3 in *SlEIN2*-silenced was very closed to it in GM fruits. As ERF2 was also subjected to the effect of EIN2 (Fig 5J), it might have led to the low ACO3 level in VIGS fruits. APETALA2a (AP2a) is another direct target of *RIN* and positively regulated by this transcription factor [41]. *AP2a* negatively regulates ethylene biosynthesis and signal [50], indicating that the accumulation of ethylene can be self-promoted and self-limited through ethylene signal pathway. The level of this gene decreased more than five-fold (Fig 5K) as RIN reduced.

### Silencing *SlEIN2* has an effect on chlorophyll binding proteins

Pathway enrichment unraveled the influence of *EIN2* silencing in LHCPs (see the link in S3 Table). The important Photosystem II (PSII) LHCP named CP29, which is located in the core antenna of PSII, has the highest Chl-a/b binding ratio [37]. The expression of *CP29* increased nearly two-fold in pTRV-*SlEIN2* tomatoes compared to OR fruits, but no significant difference between VIGS and GM fruits (Fig 5M). Two minor antenna proteins, CP24 and CP26, affect the interactions between PSII subunits [51]. The mRNA level of CP26 were similar to those of CP29 (Fig 5L). Tomato tolerance to continuous light (CL), provided by CAB13, contributes to the increase in substantial yield, as CL influences carbohydrate metabolism and photosynthesis [52], and *SlEIN2* silencing led to a larger increase in the expression of *CAB13* (Fig 5N) than in normal green fruits. GOLDEN-LIKE (GLK) and SGR are not LHCPs but also contribute to the photosynthetic capacity, and SGR can directly interact with LHCPII, a family of LHCPs belonging to PSII, separating the assembled LHCPII and leading to Chl degradation and plant degreening [39]. The SGR1, another target of RIN [41], also has physical interaction with phytoene synthase 1 (PSY1) and promotes the biosynthesis of carotenoids in tomato [53]. The expression of *SGR1* was significantly reduced in *SlEIN2*-silenced fruits, indicating its positive regulation by ethylene (Fig 5P). Chloroplast development requires GLKs and the expression of *GLK2* is typically higher than that of *GLK1*, especially in fruit shoulder [54]. The *SlEIN2*-silenced group presented higher levels of GLK2 than GM and OR fruits (Fig 5O). However, RNA-seq and relative expression analyses indicated that *SlEIN2* and ethylene act in Chl degradation and negatively regulate photosynthesis.

### Silencing *SlEIN2* leads to lycopene reduction and cell wall decomposition

*PSY*, *phytoene desaturase* (*PDS*), and *ζ-carotenedesaturase* (*ZDS*) are three of the genes involved in carotenoid metabolism and are closely related to the synthesis of lycopene. Although many *PSY* genes generate phytoene in tomato fruit, *PSY1* has a direct interaction with SGR1 and thus was analyzed here. The results showed that these genes were severely inhibited in SlEIN2-silenced fruits, particularly *PSY1*, whose expression was lowered by, at least, 90% (Fig 5Q–5S). Repressing *PDS* is sufficient to cause low lycopene content and prevent tomato from reddening [55]. Pectin methylesterase isoenzyme (PMEU1) is a ubiquitously expressed pectinesterase contributing to harden the cell wall, and the reduction of PMEU1 in fruit enhances softening rate [56]. The *SlEIN2*-silenced fruits presented an increased expression of *PMEU1*, although not as high as that of GM fruits (Fig 5T). Polygalacturonase *2A* (*PG2A*) and pectin lyase (*PL1*) are two enzymes related to pectin degradation. Both were expressed in higher amounts than *Actin* in ripening fruits, but *SlEIN2*-silencing caused their extreme reduction (Fig 5U and 5V). Although its relative expression was not tested, α-Expansin 1 (EXP1) (Solyc06g051800), which is also a booster for cell wall degradation, had a -3.38-fold change in TRV-OR/TRV-*SlEIN2* according to the RNA-seq data (S2 Table). Previous studies have also reported that RIN was associated with the promoters of *PG2a* and *EXP1*, and might positively regulate their expression [41,48]. The results obtained here showed that *SlEIN2* regulated fruit softening by affecting a series of softening-associated genes.

### Silencing *SlEIN2* altered the mRNA level of several genes involved in defense and aroma compounds generation

An in vitro study reported that tomato stress responsive factor 1 (TSRF1) interacts with the GCC-box and activates the expression of *pathogenesis-related* (*PR*) genes, to strengthen the resistance of tomato to *Ralstonia solanacearum*, and the study showed that Solyc09g089930 is upregulated by ethylene [57]; however, according to the ementary file of tomato genome research [58], this upregulation is gradually decreased during ripening. Silencing *SlEIN2* increased *TSRF1* expression and the expression of the *PR* genes *PR5* and *PR10* was also enhanced (Fig 5W and 5X). Although not tested, expression of other defense-related genes, such as *respiratory burst oxidase*, the *defense-related WRKY1* Solyc06g066370, and the *RIN4* Solyc09g059430 were also enhanced, as they were detected in KEGG pathways and RNA-seq data. The above results suggested that *SlEIN2* is involved in transformation of tomato pathogen-defense.

The genes *13-lipoxygenase (LOXC)*, *hydroperoxide lyase*, and *alcohol dehydrogenase 2* (*ADH2*) are known to participate in volatiles’ biosynthesis. Their RNAs increase during ripening [24] and ADH2 is a direct RIN target [41]. Silencing *SlEIN2* decreased the levels of both ADH2 and LOXC (Fig 5a and 5Z), thereby suggesting that this gene dowregulates the genes involved in aroma biosynthesis.

## Conclusions

**Fig 6:**
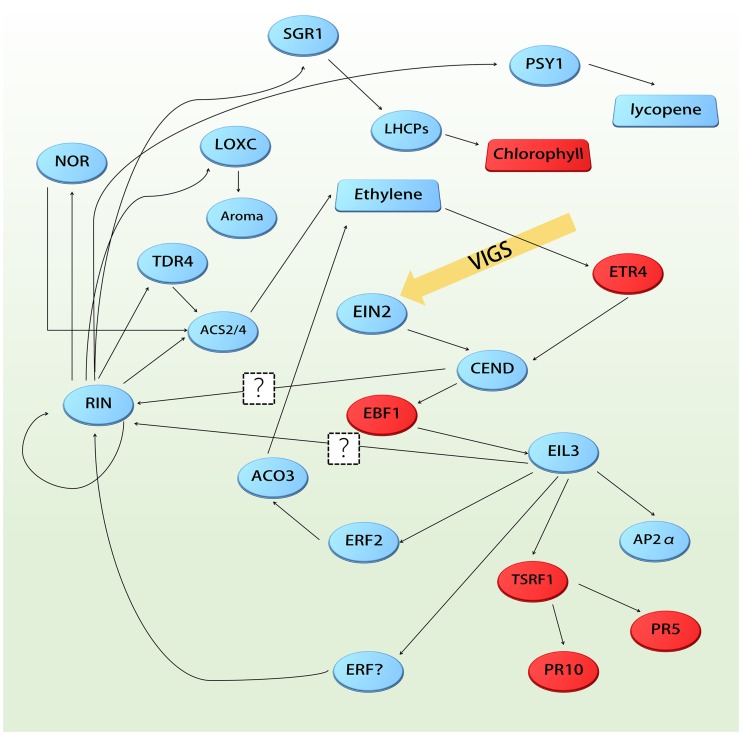
Regulation route in *SlEIN2*-silenced fruits. Red represents upregulated substances and proteins, and blue downregulated substances and proteins.

The technique, VIGS, used in the present study is a convenient and powerful tool for targeted gene silencing in tomato, producing the non-ripening phenotype in a short period. In the present study, we assessed *SlEIN2* effects on fruit ripening inhibition and this is presented in Fig 6. Silencing *SlEIN2* leads to the reduction of *RIN* in a feedback regulation process, which is generally found upstream of the ethylene signal pathway. In addition, silencing *SlEIN2* can decrease the degradation of LHCPs and chlorophyll by reducing *RIN* expression and that of its target, *SGR1*. As a result, few SGR1 proteins will interact with *PSY1* inhibiting lycopene biosynthesis, which is the reason why tomato does not turn red. Downstream regulation of *SlEIN2* is conducted by *ERFs* and silencing *SlEIN2* altered the expression of several defense-associated genes. Although *SlEIN2*-silenced and GM fruits are similar in appearance, there are significant differences in their secondary metabolites and in antenna-Chl proteins expression.

## Supporting Information

S1 Table Oligonucleotide primers used in the study. (DOCX)

S2 Table Selected differential expressed genes of control vs treatment. (XLSX)

S3 Table The results of KEGG pathway enrichment. (XLSX)

S1 Fig Gene Association of GO enrichment analysis. (PDF)
