# Supplementary material for: Transcriptome Analysis Provides a Preliminary Regulation Route of the Ethylene Signal Transduction Component, SlEIN2, during Tomato Ripening
Source: PLoS One. 2016 Dec 14;11(12):e0168287. doi: 10.1371/journal.pone.0168287 (PMC5156437; doi:10.1371/journal.pone.0168287)
Supplement: S1 Table — (DOCX) [file pone.0168287.s001.docx]

**S1 Table**

**Oligonucleotide primers used in the study.**

| **Primer** | **Sequence** |
| --- | --- |
| VIGS-*LeEIN2*-F | TAAGGTTACCGAATTCCCTGAATTGGAGCTGTAC |
| VIGS-*LeEIN2*-R | GCTCGGTACCGGATCCTGGAAATGTCCCTGTAGG |
| TRV-RNA2-F | TGTTTGAGGGAAAAGTAGAGAACGT |
| TRV-RNA2-F | TTACCGATCAATCAAGATCAGTCGA |
| CP-F | CTGACTTGATGGACGATTCTT |
| CP-R | TGTTCGCCTTGGTAGTAGTA |
| qPCR-*EIN2*-F | CTCAACCGTCTTCAGGGCATAG |
| qPCR-*EIN2*-R | GTGGCGGCAAGTTTCCATTAG |
| qPCR-*Actin*-F | CAGCAGATGTGGATCTCAAA |
| qPCR-*Actin*-R | CTGTGGACAATGGAAGGAC |
| qPCR-*ACS2*-F | GAAAGAGTTGTTATGGCTGGTG |
| qPCR-*ACS2*-R | GCTGGGTAGTATGGTGAAGGT |
| qPCR-*ACS4*-F | CGTGTAGTAATGGCTGGTGG |
| qPCR-*ACS4*-R | AGGTCCCTATTAAATCCTGGGT |
| qPCR-*ACO1*-F | ACAAACAGACGGGACACGAA |
| qPCR-*ACO1*-R | CTCTTTGGCTTGAAACTTGA |
| qPCR-*ACO3*-F | TTTCTGCAGCTCTTGTCAAATGAC |
| qPCR-*ACO3*-R | TGTTATGGAGGTAACTAGTGTGGT |
| qPCR-*RIN*-F | GGAACCCAAACTTCATCAGA |
| qPCR-*RIN*-R | TTGTCCCAAATCCTCACCTA |
| qPCR-*TDR4*-F | TCGAAGAAGGTGAAGGAGAGG |
| qPCR-*TDR4*-R | CTTGCTGCTGTGAAGAACTACC |
| qPCR-*NOR*-F | GGATTCATCAACCGGGACAC |
| qPCR-*NOR*-R | CGAATATTGCCTTAGCAGGGAG |
| qPCR-*HB1*-F | TTTCCGAGGACCAAGATCGAC |
| qPCR-*HB1*-R | TTCTGTCTCAAAGCTCTTCTCCA |
| qPCR-*ETR4*-F | AGCAATAATCATTCTCAGCCAG |
| qPCR-*ETR4*-R | TCTCCTTGCATCAACTGAACC |
| qPCR-*EBF1*-F | ATTGCCATCACTGACATAGC |
| qPCR-*EBF1*-R | AGTTATAGCAAGCGACCTC |
| qPCR-*ERF2*-F | CGTTTGTCATCCACCGACCT |
| qPCR-*ERF2*-R | GTCACGAATTTCAGCAGCCC |
| qPCR-*AP2a*-F | GAACTTAACTCAACCGAATGTACTG |
| qPCR-*AP2a*-R | CATTTCTTGGCTTGAACCTCCT |
| qPCR-*EIL3*-F | CACGAGCCTCTCTTCTTCTTAC |
| qPCR-*EIL3*-R | ACCGGCTTATGCTCAACTTC |
| qPCR-*CP29*-F | TGCCGGAAAGGTTGAGTTGG |
| qPCR-*CP29*-R | TGTTGTAATGGAGAATGGGAGTGG |
| qPCR-*CP26*-F | CTCCATCTCCATCCTCCTCTG |
| qPCR-*CP26*-R | TTCTGTCAGGTCCGTACCAC |
| qPCR-*CAB13*-F | CTCTTGAGGTTATCCATGGGAG |
| qPCR-*CAB13*-R | TTGCCCAAATAGTCCAGCC |
| qPCR-*SGR1*-F | GGGAACTTTGACTACTTCTCTAGTG |
| qPCR-*SGR1*-R | CCTTGCCACAGGTACTATGG |
| qPCR-*GLK2*-F | TCAAAGTTGACACAGTTGCC |
| qPCR-*GLK2*-R | CACACTGTCAATTGATGGAGGT |
| qPCR-*PG2*-F | AGGCTTTGGATTGCTTTTGA |
| qPCR-*PG2*-R | AGAAGGTTAAGGCCGTTGGT |
| qPCR-*PMEU1*-F | GTTCGACGCAACTACTATGCAG |
| qPCR-*PMEU1*-R | GAGTGTCTCGTCCATCGTCTC |
| qPCR-*PL1*-F | GTTTAGCAAAGAGGTTACAAAGCAC |
| qPCR-*PL1*-R | AAGATTGGCTACTAATGAAGACGG |
| qPCR-*PSY1*-F | ATGTCTGTTGCCTTGTTATG |
| qPCR-*PSY1*-R | TTCCACCACCTCTATTGATT |
| qPCR-*PDS*-F | CATTGATTATCCAAGACCAGAG |
| qPCR-*PDS*-R | CCAGCAATAACAATCTCCAA |
| qPCR-*ZDS*-F | TCTTGCTGGCTCATATACA |
| qPCR-*ZDS*-R | AGACTCAACTCATCAGATAGG |
| qPCR-*TSRF1*-F | GAAGAAACAACATCCGAAACAGTC |
| qPCR-*TSRF1*-R | TCGTAGAATCTCTTATCTCCGCTG |
| qPCR-*PR5*-F | GGTCCTACCGATTTGTCAAGATTT |
| qPCR-*PR5*-R | GTATTTGTAGGAATCTCCAATGGGA |
| qPCR-*PR10*-F | AACTTTGTTGAAGGTGGTCCA |
| qPCR-*PR10*-R | TGTCTTGTAAACACAACCTCCA |
| qPCR-*MYC2*-F | TATTCCTTCACCGGAGTCCAG |
| qPCR-*MYC2*-R | ACCCGAAACAAACGAAAGCA |
| qPCR-*TomloxC*-F | GTGCAAATACCATTAAGGCTGTG |
| qPCR-*TomloxC*-R | AGTCCAGTCTTATGATCAAGCTC |
| qPCR-*ADH2*-F | ATGTGTCCATGATGGCTGGG |
| qPCR-*ADH2*-R | GGTGATGATGCAACGAAGGC |
