# Supplementary material for: Transcriptome Analysis Provides a Preliminary Regulation Route of the Ethylene Signal Transduction Component, SlEIN2, during Tomato Ripening
Source: PLoS One. 2016 Dec 14;11(12):e0168287. doi: 10.1371/journal.pone.0168287 (PMC5156437; doi:10.1371/journal.pone.0168287)

TRV-GM VS TRV-*LeEIN2*

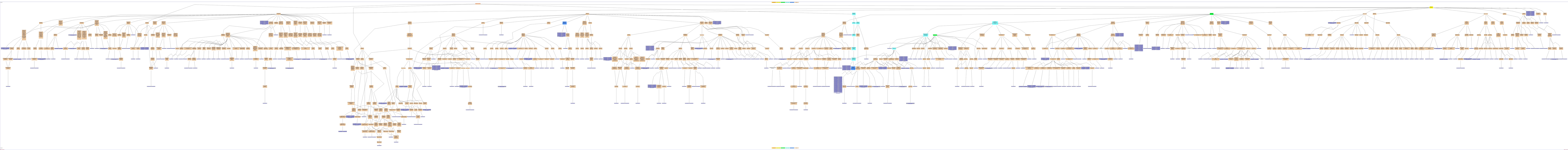

TRV-OR VS TRV-*LeEIN2*

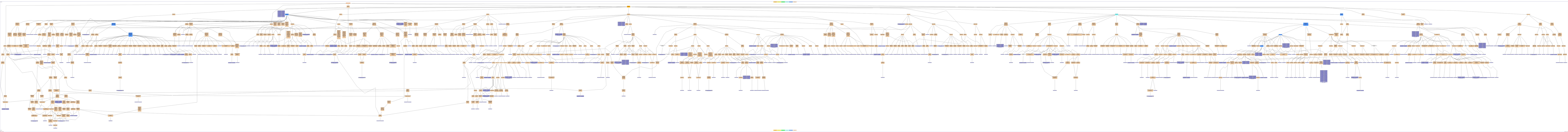

Supplement: S1 Fig — (PDF) [file pone.0168287.s004.pdf]
